# Prevalence of dual-donating amines in key regions of functional RNAs

**DOI:** 10.1261/rna.080624.125

**Published:** 2026-01

**Authors:** Andrew J. Veenis, Md. Sharear Saon, Philip C. Bevilacqua

**Affiliations:** 1 Department of Chemistry, Pennsylvania State University, University Park, Pennsylvania 16802, USA; 2 Center for RNA Molecular Biology, Pennsylvania State University, University Park, Pennsylvania 16802, USA; 3 Department of Biochemistry and Molecular Biology, Pennsylvania State University, University Park, Pennsylvania 16802, USA

**Keywords:** cheminformatics, structural bioinformatics, hydrogen bonding, noncovalent interactions, exocyclic amine

## Abstract

RNA performs many critical functions, nearly all of which are enabled by complex H-bonded structures. Nucleotides possess far fewer H-bond donors than acceptors, and the exocyclic amine is the only functional group that can donate two H-bonds, suggesting a specialized role. To assess the prevalence and structural contexts of dual-donating amines within structured RNAs, we created a computational workflow that mines and analyzes experimental RNA-containing structures. We evaluated H-bonding in over 250,000 amines from more than 1800 structures. Dual-donating amines were found most frequently in G's where they regularly interacted with diverse pairs of acceptors. In contrast, the dual-donating amines of A's and C's were less frequent, and they interacted with a more select set of acceptors. For all three nucleobases, amines that were dual-donating had both reduced solvent accessibility and higher atom density relative to amines that were single- or non-donating, indicating a tendency of dual donors to be more buried and help compact the RNA. Analysis of RNA pseudotorsion angles revealed that dual-donating amines are enriched in two non-A-form conformations, both of which are present in S-motifs found in the sarcin–ricin loop of rRNA. We find that dual-donating amines populate additional structural motifs including the GNRA tetraloop-receptor complex, the kink-turn, and the WC/H A-minor motif, which are present in the self-splicing group I intron, the SAM riboswitch, and the poly(A)-bound ENE. We suggest that dual-donating amines may enhance interactions by reducing conformational entropy loss of the RNA as well as strengthening nearby H-bonds.

## INTRODUCTION

RNA serves myriad functions in the cell. It is involved in splicing, tRNA processing, and translation and does so by adopting complex structures (Leamy et al. 2016). These structures often contain bulges, loops, and junctions that can interact by H-bonding, π-stacking, and metal coordination. Such interactions can compact the RNA, leading to function.

One method of assessing interactions across RNA-containing structures is cheminformatics, which entails mining and analyzing molecular data. The Protein Data Bank (PDB; rcsb.org) contains a wealth of data on structured RNAs, with 8595 RNA-containing entries as of April 10, 2025 (Berman et al. 2000). Over the last decade, the number of high-resolution RNA structures has increased dramatically, a trend that should continue given the interest in RNA and ongoing improvement in RNA structure determination methods (Ma et al. 2022). Using cheminformatic approaches, these structures can be surveyed using Python along with tools such as PyMOL, Biopython, and GEMMI (Hamelryck and Manderick 2003; Cock et al. 2009; Wojdyr 2022). Indeed, our group has contributed to cheminformatics of RNA by identifying the prevalence of *syn* bases in the active sites of RNAs (Sokoloski et al. 2011), determining common catalytic strategies of small ribozymes (Seith et al. 2018), and reporting alternative protonation in the formation of RNA structures (Saon et al. 2025). Knowledge of the underlying RNA chemistry, such as bond distances, angles, molecular geometries, and intermolecular interactions, drives the design of computational workflows to analyze the structural data. Combining information extracted from hundreds of unique structures, leading to hundreds of thousands of interactions, enables a deeper understanding of RNA interactions and how they might promote RNA form and function.

**FIGURE 1.**
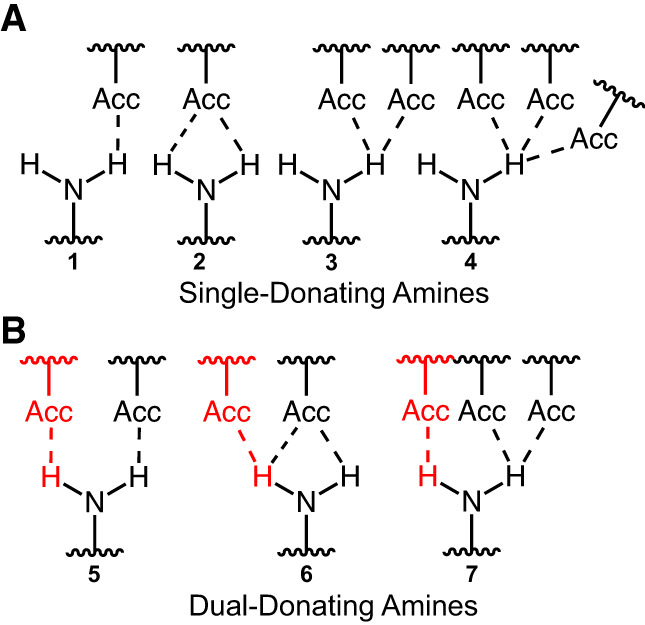
H-bonding possibilities for an exocyclic amine. Seven scenarios are depicted, demonstrating amines that are (*A*) single- or (*B*) dual-donating. Single-donating scenarios 1, 2, and 3 can become dual-donating by gaining the red H-bonds depicted in scenarios 5, 6, and 7, respectively. In an effort to be comprehensive, diverse scenarios are considered even though not all of them (e.g., 2 and 6) can occur given our H-bond criteria. Bifurcated H-bond examples are present in scenarios 2, 3, 6, and 7, and a trifurcated H-bond example is present in scenario 4.

Herein, we applied a cheminformatic approach to study dual-donating amines found within structured RNAs. The exocyclic amines of A's, C's, and G's were classified as non-, single-, or dual-donating as a function of how many H-bonds they donate. Three different scenarios can yield amines that are classified as single-donating (Fig. 1A): (1) amines that donate one H-bond, (2) amines that donate two H-bonds where both involve the same acceptor, and (3) amines that donate two or more H-bonds where all H-bonds involve the same H. While the latter two scenarios technically involve more than one H-bond, the capacity of the amine to donate two “separate” H-bonds is not utilized; rather, the multiple H-bonds arise through H-bond bifurcation or trifurcation (Steiner 2002; Feldblum and Arkin 2014). We considered dual-donating amines to be those that donate two or more H-bonds where the two amine H's and two or more different acceptors are involved (Fig. 1B).

Our analysis of these amines includes evaluations of (1) nucleobases of dual-donating amines and their H-bond acceptors, (2) dual-donating amine burial within their encompassing structures, and (3) correlations between amine dual-donation and RNA conformations. We consider more than 1800 RNA-containing PDB entries enumerated in the 3.386 release of the actively maintained Representative Sets of RNA 3D Structures, which reduces redundancy relative to all available PDB entries (Leontis and Zirbel 2012). Because many PDB structures lack H's, our workflow adds these to the exocyclic amines and then identifies H-bonds on the basis of H-acceptor distance and nitrogen–H-acceptor angle criteria. The amines are then sorted into “non,” “single,” and “dual” categories. When combined with other structural data, the results reveal dual-donating amine prevalence and context.

## RESULTS

**FIGURE 2.**
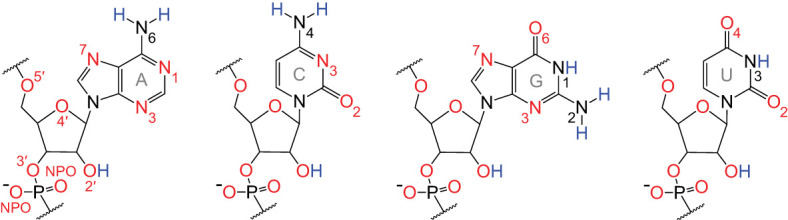
H-bond acceptors vastly exceed donors in RNA. Acceptors and donors are depicted in red and blue, respectively. Oxygens can accept two H-bonds, while unprotonated nitrogens can accept a single H-bond. The exocyclic amine, which cannot accept an H-bond due to donation of its lone electron pair into the ring system, can donate two H-bonds. In contrast, all other nitrogen and oxygen donors can donate only a single H-bond. This counting of H-bonds does not consider multifurcated interactions (e.g., a bifurcated H-bond), which are mentioned in Figure 1. Labels to the key atoms are provided.

RNA possesses many more H-bond acceptors than donors (Fig. 2). Among the four canonical RNA nucleotides, there are 62 lone electron pairs from oxygen and nitrogen atoms, which can each accept an H-bond, while there are only 12 polar H's that can donate one. Thus, acceptors outnumber donors 5:1. Notably, all 28 oxygen atoms in RNA have two lone electron pairs and so can accept multiple H-bonds; in contrast, only the three exocyclic amines, one present in each of A, C, and G, can donate multiple H-bonds.

### Amine-acceptor geometries inform H-bond selection criteria

**FIGURE 3.**
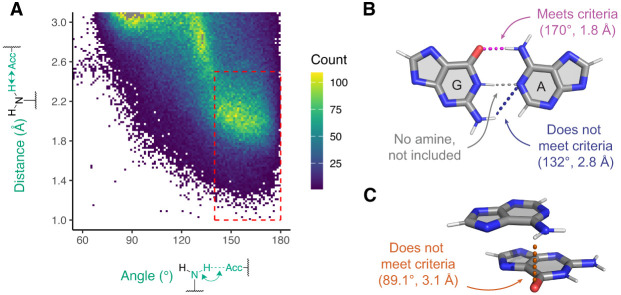
Amine-acceptor pair measurements to determine H-bonding criteria. (*A*) Heat map depicting amine-acceptor geometries. Because of their dominance from canonical base pairs, A(N6)–U(O4), C(N4)–G(O6), G(N2)–C(O2), and G(N2)–C(N3) pairs are not included. Any pairs with distance and angle measurements that fall within the dashed red box are considered H-bonding. The points colored gray have counts beyond the maximum of the color scale. (*B*,*C*) Examples that meet and do not meet criteria. The angle and distance are shown for each interaction that involves an amine. (*B*) Depiction of a GA base pair from chain a in PDB ID 7UW1 involving G91 and A76 (Morgan et al. 2023). Magenta: amine interaction that falls within the dashed red box. Blue: amine interaction that falls outside the dashed red box. Gray: an interaction that does not involve an amine and thus is not included. (*C*) Depiction of a stacked AG from the same chain involving A1173 and G1174. Orange: amine interaction that falls outside the dashed red box.

We began studying dual-donating interactions with a cheminformatics approach. The 3.386 release of the Representative Sets of RNA 3D Structures, hereafter referred to as the “Representative Data Set,” lists RNA-containing structures that can be downloaded from the PDB and affords over 250,000 amines. Our algorithm queried these amines for H-bonds to nearby acceptors. This was accomplished by comparing the measured H-bond distance and angle to criteria informed by a heat map of amine-acceptor H-bonding geometries (Fig. 3A). In this heat map, the two key geometries considered are (1) the distance between the acceptor and an amine's H (added using PyMOL) and (2) the angle between the nitrogen, an amine H, and the acceptor. To assess these criteria, acceptors near each amine were identified, and their distances and angles were plotted as a heat map (Fig. 3A). Because interactions from canonical base-pairing would overwhelm the plot, A(N6)–U(O4), C(N4)–G(O6), G(N2)–C(O2), and G(N2)–C(N3) pairs were excluded from this plot, although they are included in all subsequent analyses. Individual heat maps of each of these four amine-acceptor pairs are presented in Supplemental Figure S1.

The resulting heat map revealed a major population toward the middle-right region colored in yellow and green (Fig. 3A). The edges of this population guided the selection of the H-bond distance and angle criteria of ≤2.5 Å and ≥140°, which is represented by the dashed red box. Many of the amine-acceptor pairs in the populations outside of the dashed red box correspond to weak interactions such as where amines are positioned near acceptors due to other, stronger interactions. For example, in a GA base pair, the G(N2)–A(N1) exhibits a geometry of 2.8 Å and 132° (Fig. 3B). Another example is a stacked AG where the A(N6)–G(O6) exhibits a geometry of 3.1 Å and 89.1° (Fig. 3C).

### Preferences for nucleobase amine hydrogen bonding

**FIGURE 4.**
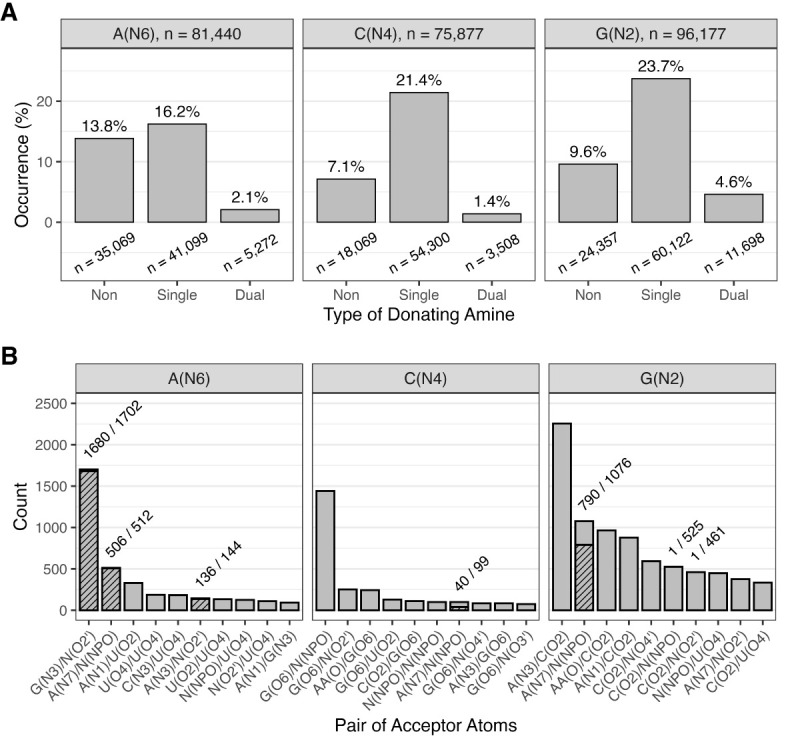
Prevalence of H-bonding interactions among amines. (*A*) Percentage of non-, single-, and dual-donating amines among all A's, C's, and G's. The nine columns sum to 100%. The number of amines surveyed are provided above the plots, and the number belonging to each donor type are provided below its respective column. (*B*) Number and identity of acceptor pairs that are engaged with dual-donating amines. Hatched portions of columns represent the number of acceptor pairs that are part of the same residue, and the numbers above these columns indicate the fraction of pairs belonging to the same residue. When the acceptor is a backbone atom, canonical RNA and amino acid residues are denoted with “N” and “AA,” respectively. The top 10 acceptor pairs per nucleobase amine are provided. Select examples of these interactions are provided in Figure 5 and Supplemental Figure S2.

A first step for understanding the role of dual-donating amines in RNA structure is evaluating the frequency at which A, C, and G amines dual donate. We assessed the structural data on H-bonding of the more than 250,000 amines in the Representative Data Set, with more than 75,000 amines for each nucleobase (Fig. 4A). We considered all possible polymer acceptors, including RNA, DNA, and amino acids. In contrast to Figure 3, no amine-acceptor measurements that met the H-bond criteria were excluded. Overall, dual-donating amines are relatively rare, making up only 8.1% (2.1% A + 1.4% C + 4.6% G) of our data set. (This result depends on the H-bond criteria, as looser criteria would increase the fraction of dual-donating amines.) Nonetheless, there are still many dual-donating amines in the Representative Data Set, with 20,478 instances (5272 A's + 3508 C's + 11,698 G's). Notably, G's are highly represented, containing more dual-donating amines than A's and C's combined. This enrichment holds even when considering the relatively higher number of G's in the Representative Data Set (96,177 G's vs. 81,440 A's and 75,877 C's), leading to 1-in-8 G's, 1-in-15 A's, and 1-in-22 C's being dual-donating. The amine of G resides in the minor groove, while the amines of A and C are in the major groove. The minor groove of A-form RNA is wide and shallow, affording greater accessibility to the amine of G, which may account for some of its enrichment.

Single-donating amines are the largest proportion of the distribution at 61.3% (16.2% A + 21.4% C + 23.7% G) (Fig. 4A), likely from the abundance of canonical base pairs. The enrichment of C and G might reflect the bias of the structural biology community to study strongly folding RNAs. Finally, nondonating amines account for the remaining 30.5% (13.8% A + 7.1% C + 9.6% G) of all amines, with an enrichment in A, which may reflect the above biases.

**FIGURE 5.**
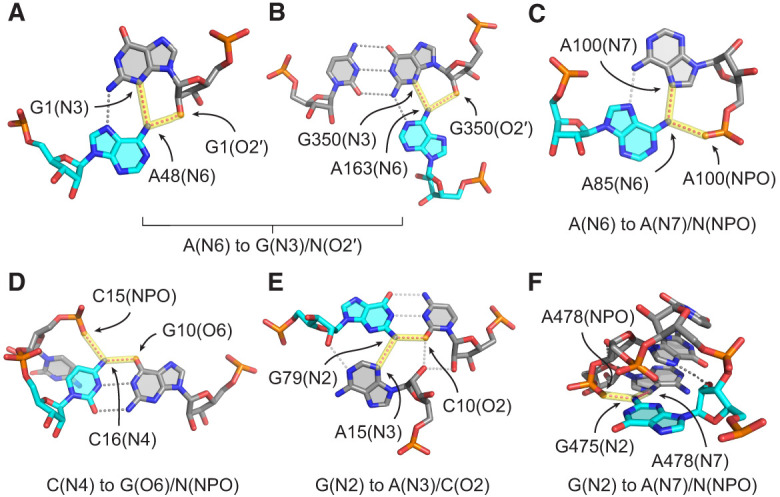
Common acceptor examples for dual-donating amines. (*A*,*B*) Adenine dual-donating amines interacting with the N3 and O2′ of the same G forming (*A*) a sheared GA base pair (PDB ID 3FS0, chain A, A48) (Pitt and Ferré-D'Amaré 2009) and (*B*) a WC/H A-minor motif (PDB ID 7QCA, chain L50, A163) (McLaren et al. 2023). (*C*) A85(N6) dual donates to the N7 and NPO of A100 (PDB ID 9AXU, chain 2, A85) (Gemin et al. 2024). (*D*) C16(N4) dual donates to a G(O6) in a canonical GC base pair and an NPO from a different residue (PDB ID 7B9V, chain 6, C16) (Wilkinson et al. 2021). (*E*) G79(N2) dual donates to a C(O2) in a canonical GC base pair and an A(N3), forming a traditional type I A-minor motif (Baulin 2021) (PDB ID 8A22, chain A5, G79) (Tobiasson et al. 2022). (*F*) G475(N2) dual donates to the N7 and NPO of A478 within a GNRA tetraloop (PDB ID 6ERI, chain AA, G475) (Perez Boerema et al. 2018). In all six panels, magenta dots highlighted in yellow depict H-bonds from dual-donating amines, and gray dots represent other H-bonds. All instances of these dual-donor-to-acceptor-pair interactions are provided within CSV and Python files; see Supplemental Table S1 and Supplemental Material.

We turn our focus to the identity of the atom pairs that accept the H-bonds from the dual-donating amines. Starting with dual-donating A(N6), it is striking that the three top acceptor pairs do not involve U(O4), the acceptor in a canonical AU base pair (Fig. 4B). Apparently, most A dual-donating amines do not simply recruit an additional H-bonding acceptor to an AU pair. The dominant acceptor pair is G(N3)/N(O2′), and in 1680 out of 1702 cases these acceptors belong to the same G, for example in a sheared GA base pair (Fig. 5A). Sheared GA base pairs form when the Hoogsteen edge of an A interacts with the sugar edge of a G, with the dual-donating amine providing two H-bonds and the G(N2) providing a third to this interaction (Jang et al. 2004). The sheared GA dominates the G(N3)/N(O2′) acceptor pair category (1518/1702), consistent with previous reports (Olson et al. 2019). Intriguingly, the G(N2) is also a dual donor in 186 of these 1518 sheared GAs. All instances of the interactions depicted in Figure 5 are provided within CSV and Python files (Supplemental Table S1) and can be viewed within a PyMOL session (see Materials and Methods).

Another example of the G(N3)/N(O2′) accepting pair for A(N6) is in the A-minor motif (Fig. 5B). This is one of the most prevalent motifs in structured RNAs and involves the interaction of an A with the minor groove of another pair (Baulin 2021). Intriguingly, Steitz and colleagues recently described a new subclass of A-minor motif that they coined the “WC/H A-minor” motif (Torabi et al. 2021), which involves the WCF and/or Hoogsteen edge of A interacting with the minor groove of a WCF base pair. We identified 40 instances of this base triple motif. In contrast to the sheared GA base pairs, most WC/H A-minor instances (31/40) involve G(N2) donating to A(N1) since the A(N7) is swung away from the G. Of note, the G(N2) is itself a dual-donating amine in nearly all cases (38/40).

The next most common acceptor pair for A(N6) is A(N7)/N(NPO), where NPO stands for nonbridging phosphoryl oxygen, with a count of 512 (Fig. 4B). In nearly all these occurrences (506/512), the acceptors belong to the same A. Furthermore, the accepting A donates back an A(N6)-to-A(N7) H-bond in 458 out of 506 cases (Fig. 5C), resulting in a *trans* AA base pair involving the Hoogsteen edge of both A's and at least one NPO. The amine of the second A dual donates in 75 cases, the majority of which (50 cases) feature symmetrical interactions where the amines of both A's dual donate to each other's A(N7)/N(NPO). The negative charge of the NPO acceptor likely plays a key role in stabilizing this interaction (see Discussion). The remainder of the acceptor pairs for A(N6) occurred much less frequently, with counts of 330 or less.

Moving to C(N4), the top five acceptor pairs contain G(O6) (Fig. 4B), the acceptor in canonical base pairs, deviating from A(N6). The most common acceptor pair for C(N4) is G(O6)/N(NPO), with a count of 1441, and these two acceptors never belong to the same residue (Fig. 4B). As depicted in Figure 5D, the parent C of the dual-donating amine forms a canonical base pair with the G, which occurs in most cases (1398/1441). Given the charge on the phosphate, a significant electrostatic component is expected for this interaction.

Considering G, the interactions of its amine differ from those of A and C in that the less frequent acceptor pairs still occur quite often (Fig. 4B), indicating that G's amine engages in a diverse set of dual-donating interactions. The dominant acceptor pair for G(N2), in 2256 instances, consists of A(N3)/C(O2), where the latter is the WCF H-bonding partner of G(N2) (Fig. 4B). Within most of the cases (2178/2256), the G forms a canonical pair with the C, and the sugar edge of A interacts with the minor groove of the GC pair, making this a traditional type I A-minor motif (Fig. 5E; Baulin 2021). Furthermore, 8% of these cases (179/2178) include an A that also bears a dual-donating amine.

The second most common acceptor pair for G(N2) is A(N7)/N(NPO) (Fig. 4B). Of the 1076 amines in this category, 790 donate to acceptors that belong to the same A (Fig. 5F). Inspection of a sample of this subset reveals tetraloops and pentaloops contributing to this dual-donating interaction, where the G and A are the first and last members of the loop, respectively. Twenty-three of the 790 A's that accept this dual-donating interaction bear an N6 that is also dual-donating.

Finally, we note that for both C(N4) and G(N2), the third most prevalent acceptor pair consist of a peptide backbone carbonyl oxygen, AA(O), and the canonical base pair acceptor (Supplemental Fig. S2). For C(N4), the AA(O) is in the major groove, while for G(N2), it is in the minor groove, making the dual-donating amine versatile. Like the phosphate backbone, the backbone carbonyl oxygen has significant negative charge, which could also strengthen these H-bonds (see Discussion). Notably, no amino acid acceptor was present in the top 10 acceptor pairs for A(N6). Evidently, dual-donating amines belonging to C and G play a greater role in protein binding. All instances of the interactions depicted in Supplemental Figure S2 are provided within CSV and Python files (Supplemental Table S1) and can be viewed within a PyMOL session (see Materials and Methods).

### Dual-donating amines tend to be buried in solvent inaccessible and dense regions

Owing to long-range interactions, highly structured RNAs are often globular-like, in which some residues are located closer to the interior. We were curious whether amine dual donation, being a multivalent interaction, was correlated with RNA compaction. We thus measured the solvent accessibility of the exocyclic amines and the density of their surrounding atoms.

**FIGURE 6.**
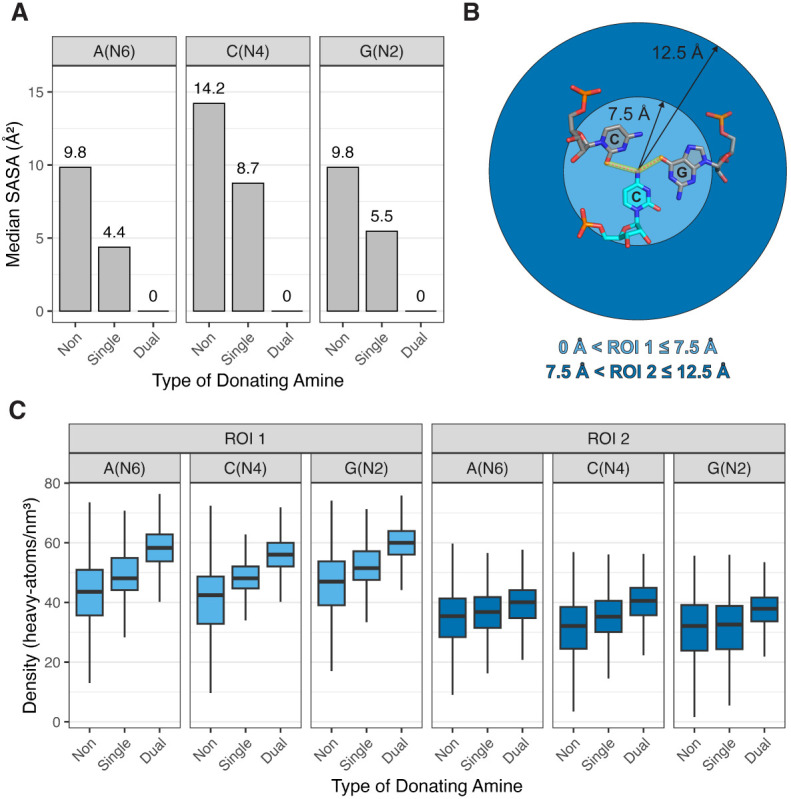
Amine solvent accessibility and surrounding density. (*A*) Median SASA values for nitrogens of non-, single-, and dual-donating amines from each type of nucleobase. Corresponding box plots are provided in Supplemental Figure S3A. (*B*) Diagram depicting the two regions of interest, ROI 1 (light blue) and ROI 2 (dark blue). The regions are centered about the amine nitrogen of interest, which in this example is C1092(N4) (PDB ID 1MMS, chain C) (Wimberly et al. 1999). (*C*) Density of heavy atoms within ROI 1 and ROI 2. All adjusted *P*-values for heavy atom density are <10^−5^ for the differences observed in the distributions within each of the six subplots (see Materials and Methods). Outliers were omitted from these plots but are included in Supplemental Figure S3C.

We began by considering solvent accessible surface area (SASA), which measures exposure of a residue or atom to solvent. We used the Biopython suite of tools, which implements an algorithm based on that developed by Shrake and Rupley (1973). In all cases, we measured the SASA of the nitrogen of the amine, without H's present. We found that the median SASA values for the amine nitrogens of A, C, and G decreased along the series non-, single-, and dual-donating, where it fell to 0 Å^2^, indicating that dual donors are fully buried (Fig. 6A; Supplemental Fig. S3A). The nitrogens of A's and G's exhibited similar median SASA values of 9.8 and 4.4–5.5 Å^2^ for non and single donors, respectively. In contrast, the values were elevated for C(N4), at 14.2 and 8.7 Å^2^, which may be due to the absence of the imidazole ring, which can stack and whose N7 can assist in burial. Nonetheless, the median SASA for dual-donating C(N4) is still 0 Å^2^, reinforcing the notion of dual-donating amine burial. Notably, the median SASA values of 0 Å^2^ for dual-donating amines from all three bases indicate crowding not just along the edges of the donating base, but above and below it as well. The importance of atoms above and below was confirmed by omitting all atoms other than the residues involved in a dual-donating interaction—we chose the central C(N4) within the base triple depicted in Figure 6B—and finding a sizeable SASA (10.9 Å^2^ in this example). Finally, we note that a slight fraction of dual donors has nonzero, albeit typically small, SASA values (Supplemental Fig. S3B).

We also assessed burial of the dual-donating amines by measuring the density of surrounding heavy (i.e., non-H) atoms. This quantity was measured in two regions of interest (ROIs), an inner one within 7.5 Å of the amine nitrogen, termed “ROI 1,” and an outer one between 7.5 and 12.5 Å of the amine nitrogen, termed “ROI 2” (Fig. 6B). We included heavy atoms from RNA, proteins and other polymers, but not from water, inorganic species, and organic molecules (i.e., ligands). The 7.5 Å cut-on for ROI 2 was chosen as it defines the boundary of the nucleobases in a base triple (Fig. 6B).

For the ROI 1 densities, the medians for non-, single-, and dual-donating A amines increased with values of 44, 48, and 58 atoms/nm^3^, respectively (Fig. 6C; Supplemental Fig. S3C). The medians for C and G showed the same trend, with values of 42, 48, and 56 atoms/nm^3^ and 47, 51, and 60 atoms/nm^3^, respectively. Notably, the increase in density was greater going from single- to dual-donating (changes of 10.2, 7.9, and 8.5 atoms/nm^3^ for A's, C's, and G's, respectively) when compared with going from non- to single-donating (changes of 4.5, 5.7, and 4.5 atoms/nm^3^). Additionally, the distributions for the dual-donating amines were tighter, with interquartile ranges (IQRs) averaged across the three nucleobases decreased by 10% relative to single donors and by 46% relative to nondonors. The narrowness of the distributions suggests that higher density is a consistent property of dual-donating amines.

For ROI 2, the median densities also increased upon going from non- to single- to dual-donating amines for all three nucleobases, albeit with a more moderate incline (Fig. 6C; Supplemental Fig. S3C). Moreover, again the magnitude of the increase in going from single- to dual-donating remained greater than going from non- to single-donating, and the IQRs tightened as the amine progresses from non- to single- to dual-donating. This tightening of IQR is especially prominent for G(N2), where the dual category IQR (8.0 atoms/nm^3^) was nearly half that of the single category IQR (14.5 atoms/nm^3^). It is remarkable that higher atom density and tighter IQR persist in distal amine regions 7.5–12.5 Å away.

### Glycoside bond dihedrals vary depending on amine H-bond donation

Owing to rotations available at the glycosidic bond and six sugar-phosphate backbone dihedral angles, RNA is an extraordinarily flexible polymer. Because they engage in two interactions, dual-donating amines have the potential to reduce the conformational entropy loss of the RNA upon folding, which might enhance certain conformations. We therefore investigated whether donating amines prefer certain conformations about their glycosidic bonds.

The χ dihedral reports a nucleotide's conformation about its glycosidic bond and is typically categorized as being *anti* or *syn*, with the base away from or over the sugar, respectively. Our laboratory conducted a structural survey of functional RNAs for nucleobases in the rare *syn* conformation (Sokoloski et al. 2011). An amine engaged in dual donation could, in principle, help stabilize this energetically unfavorable conformation. We thus measured the χ dihedrals of residues bearing non-, single-, and dual-donating amines.

**FIGURE 7.**
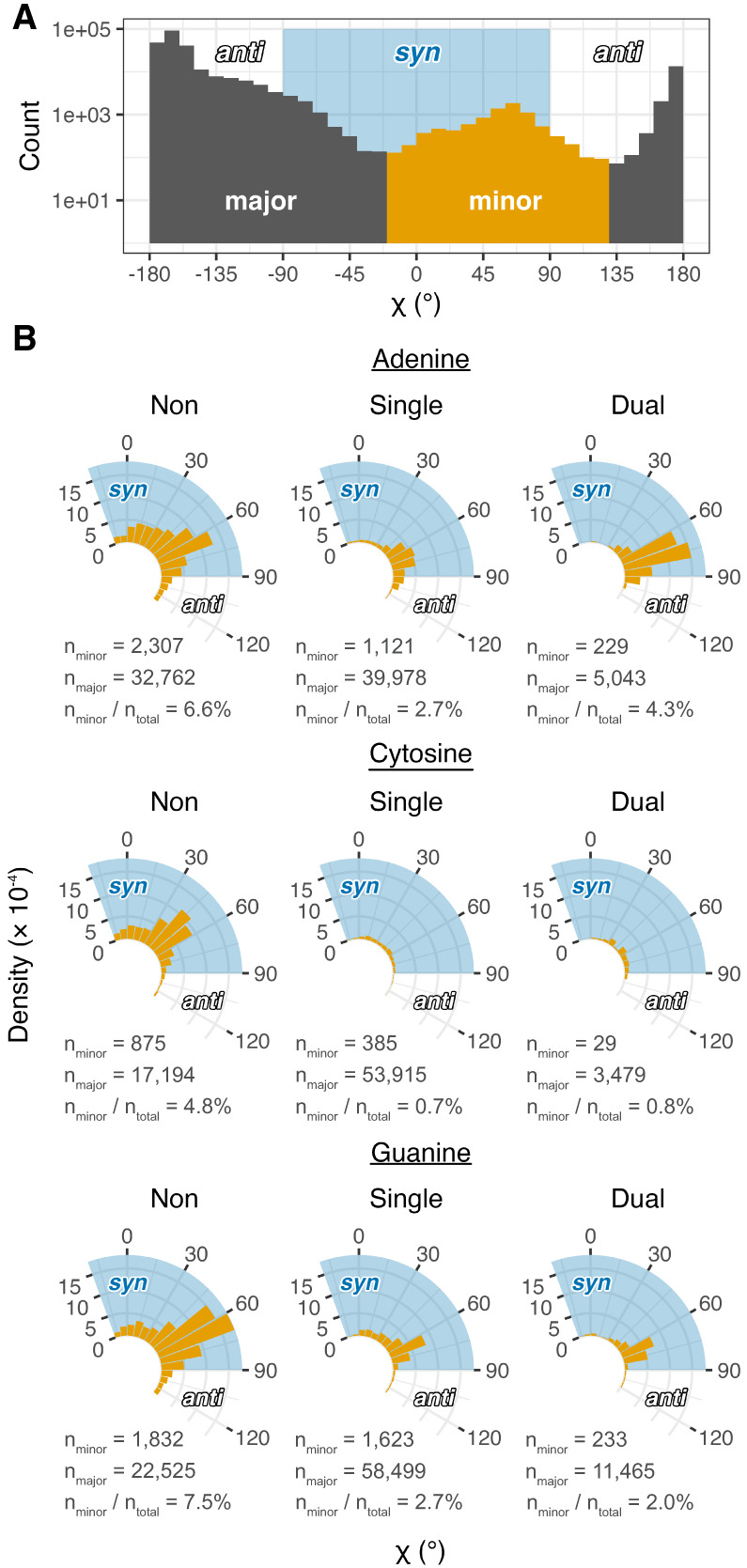
Analysis of χ dihedrals of the minor population. (*A*) Histogram (logarithmic *y*-scale) of all A, C, and G residues spanning the entire χ dihedral range. A minor population (orange bars) is primarily in the *syn* conformation (blue shading). (*B*) Radial density plots of the minor population χ dihedrals separated into non-, single-, and dual-donating amines. Densities are based on calculations of the full dihedral range presented in Supplemental Figure S4. The number of residues in the minor and major populations are provided under each plot for all nucleobase and donating amine types, along with the corresponding percentages of residues that occupy the minor population. The blue shaded regions correspond to nucleobases that adopt the *syn* conformation, which extends beyond the lower bound of these plots to −90°.

A histogram for all three bases revealed a major and a minor population, with the latter mostly corresponding to the *syn* orientation (Fig. 7A), where *syn* nucleobases have χ dihedrals from −90° to 90° (Bloomfield et al. 2000). We then prepared density plots for each nucleobase and amine donation type. This was done across the entire −180° to 180° range (Supplemental Fig. S4), with just the −20° to 130° range, corresponding to the minor population, displayed in Figure 7B.

We first focus on the minor population, given the functional importance of *syn* nucleobases (Sokoloski et al. 2011). A's with dual-donating amines are enriched when compared to those with single donors (4.3% vs. 2.7%) (Fig. 7B). For C's, however, single- and dual-donating amines are both depleted (0.7% and 0.8%). It is well known that pyrimidines adopt the *syn* conformation less frequently than purines owing to clash of the O2 with the ribose sugar (Saenger 1973, 1984; Sundaralingam 1975; Sokoloski et al. 2011). Lastly, for G's, there appears to be a slight preference for single-donating amines over dual donors (2.7% vs. 2.0%), and the dual-donating amines are intermediate in density to A's and C's. Relative to A's, the lower density of G's in the minor population may be due to a paucity of acceptors on the top face of the sugar and/or to steric clash of the G(N2). Across all three nucleobases, any type of H-bond donation by the amine disfavors the minor conformation indicated by the relatively higher densities for the nondonors (6.6% for A, 4.8% for C, and 7.5% for G). This is likely because the major conformation exposes the amine to a wider array of potential acceptors and because WCF base pairs are common and adopt the *anti* conformation.

Focusing on the major population (Supplemental Fig. S4), we note that single- and dual-donation narrow the distributions of the *anti* conformation for all three bases relative to nondonors, albeit dual-donation pushes A back toward a somewhat broader distribution. Apparently, single- and dual-donation restricts the conformational heterogeneity of the nucleosides.

### Pseudotorsional analysis reveals dual-donating amine enrichment in S-motifs

To further assess enrichment of dual-donating amines in select conformations, we next considered conformations along the backbone. For ease of analysis, the six covalent bonds linking the backbone together were reduced to two virtual bonds, one involving $P_{i}-{C4}_{i}^{\prime }$ and another involving ${C4}_{i}^{\prime }-P_{i+1}$, as described previously (Duarte et al. 2003). These virtual bonds give rise to two pseudotorsion angles, η and θ, ${C4}_{i-1}^{\prime }-P_{i}-{C4}_{i}^{\prime }-P_{i+1}$ and $P_{i}-{C4}_{i}^{\prime }-P_{i+1}-{C4}_{i+1}^{\prime }$, respectively, where *i* represents the residue of interest. This reduction allows Ramachandran-like plots to be constructed, in which different RNA motifs often occupy different regions (Keating et al. 2011).

**FIGURE 8.**
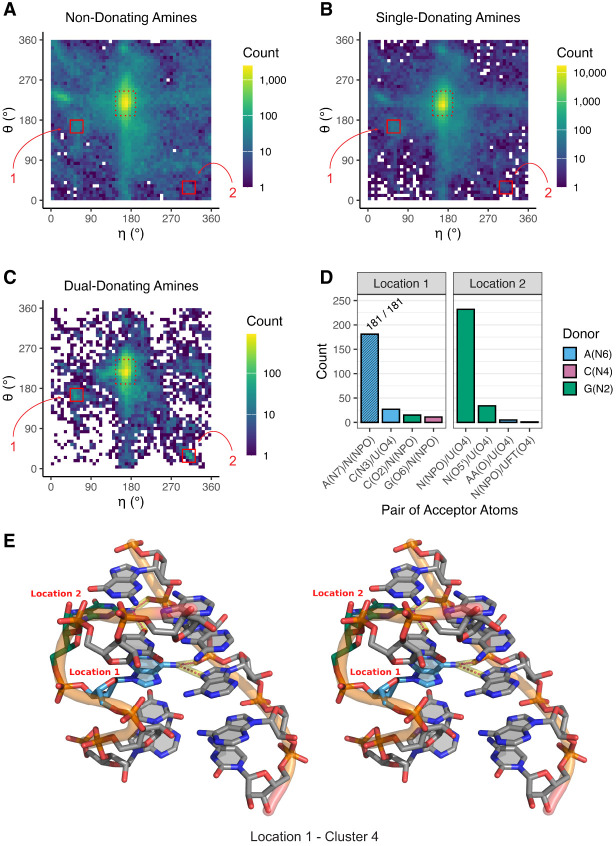
Key dual-donating amines involved in S-motifs. (*A–C*) Heat maps depicting pseudotorsion angles of residues bearing (*A*) non-, (*B*) single-, and (*C*) dual-donating amines. The color scale is logarithmic to accommodate the wide range of data. Regions that correspond to A-form helices are in dashed red boxes, and locations of interest, “Location 1” and “Location 2,” are in solid red boxes. The residues within Location 1 and Location 2 of (*C*) are provided within CSV and Python files; see Supplemental Table S1 and Supplemental Material. (*D*) Number of acceptor pairs that are engaged with dual-donating amines for Location 1 and Location 2. Symbols “N” and “AA” are used to denote any canonical RNA or amino acid residue, respectively, when the acceptor is a backbone atom. Only the top four acceptor pairs are shown for each Location. (*E*) Cross-eye stereoview of the representative structure from the dominant cluster (C4) (Supplemental Fig. S6) of the Location 1 analysis. An S-motif is depicted with an A dual-donating amine from Location 1 (blue; PDB ID 6ZU5, chain S60, A806) and a G dual-donating amine from Location 2 (green) (Ehrenbolger et al. 2020). Magenta dots highlighted in yellow depict H-bonds from the two dual-donating amines. All representative structures from all four clusters for Location 1 and Location 2 are depicted in Supplemental Figure S7.

In an effort to identify any RNA motifs unique to dual-donating amines, we plotted heat maps of the pseudotorsion angles for non-, single-, and dual-donating amines (Fig. 8A–C). Pseudotorsion conformations corresponding to A-form helices, with values of 145° > η > 190° and 190° > θ > 245°, dominate the plots as reported (Duarte and Pyle 1998; Humphris-Narayanan and Pyle 2012; Grille et al. 2023). For dual-donating amines, two additional locations with relatively high counts emerged (Fig. 8C). For non- and single-donating amines, the same two regions are not as enriched (Fig. 8A,B).

The dual donors and their acceptors in Location 1 and Location 2 were determined (Fig. 8D). For Location 1, which had η values ranging from 43° to 72° and θ values ranging from 151° to 180°, there was a single dominant interaction, that of A(N6) donating to A(N7)/N(NPO), with a count of 181. This matched the second most prevalent acceptor pair for all A dual-donating amines (Fig. 4B) where the majority of such pairs involved a *trans* base pair along the Hoogsteen edges of two A's (Fig. 5C). For Location 2, which had η values ranging from 295° to 324° and θ values ranging from 14° to 43°, there was again a single dominant interaction, here of G(N2) donating to N(NPO)/U(O4), with a count of 232. This corresponds to the eighth most prevalent acceptor pair for all G dual-donating amines (Fig. 4B). It is notable that the second most prevalent interaction, with a count of 34, was like the first, with the only difference being that the first acceptor is a bridging, rather than a nonbridging, phosphoryl oxygen. Further analysis revealed that in 83 cases, a residue in Location 1 is connected to the 5′-end of a residue from Location 2; thus, the residues from Location 1 and Location 2 are sometimes adjacent in sequence. These 83 instances, along with all other residues bearing dual-donating amines and corresponding to Location 1 or Location 2, are provided within CSV and Python files (Supplemental Table S1) and can be viewed within a PyMOL session (see Materials and Methods).

After considering plots similar to Figure 8A–D, we manually inspected a variety of structures contributing to Location 1 and Location 2. We found that many of them belong to S-motifs, which make up part of the sarcin/ricin loop in rRNA, with η and θ values close to reported values (Correll et al. 2003; Duarte et al. 2003). To identify a representative structure for each Location, we carried out a clustering analysis (see Materials and Methods). Given the known geometry of an S-motif, we first extended the sequence in both directions along the strand containing the residue of interest and then repeated this for its base-pairing partner, resulting in a 6 × 5 nucleotide fragment. This process was conducted on all 181 cases for the major acceptor pair for Location 1 and all 266 cases for the two major acceptor pairs for Location 2. The 181 Location 1 fragments were superposed, as were the 266 Location 2 fragments. Cutoffs of 3.40 Å for Location 1 and 4.10 Å for Location 2 were chosen on the basis of the resulting Silhouette scores (Supplemental Fig. S5). The resulting dendrogram had four clusters for Location 1, with the dominant cluster (C4) containing 97 of the 181 fragments, and four clusters for Location 2, with the dominant cluster (C3) containing 158 of the 266 fragments (Supplemental Fig. S6).

As expected, the representative structures (chosen as described in Materials and Methods) from the dominant clusters for Location 1 (Fig. 8E; Supplemental Fig. S7D) and Location 2 (Supplemental Fig. S7G) exhibit S-motifs, with the characteristic “S” shape seen in the left-most strand. Notably, in each example, the dual-donating amine of G in Location 2 (green) is bulged, while the dual-donating amine of A in Location 1 (blue) forms a *trans* AA base pair. The sequence 5′-AGU-3′ in the S-motif has the U stacking on the A, where the G is in Location 2 and the A in Location 1, and one of the acceptors of the dual-donating amine of G is the O4 of the U. Out of the eight clusters identified, six exhibited S-motifs: C1, C2, and C4 for Location 1 and C1, C3, and C4 for Location 2 (Fig. 8E; Supplemental Fig. S7), with the two missing clusters having relatively few fragments (Supplemental Fig. S6). Altogether, an analysis of pseudotorsion angles followed by clustering of fragments revealed that dual-donating amines are found in S-motifs at two distinct positions, with both dual donors sometimes occurring in the same structure.

### Key RNA motifs in functional RNAs contain multiple dual-donating amines

**FIGURE 9.**
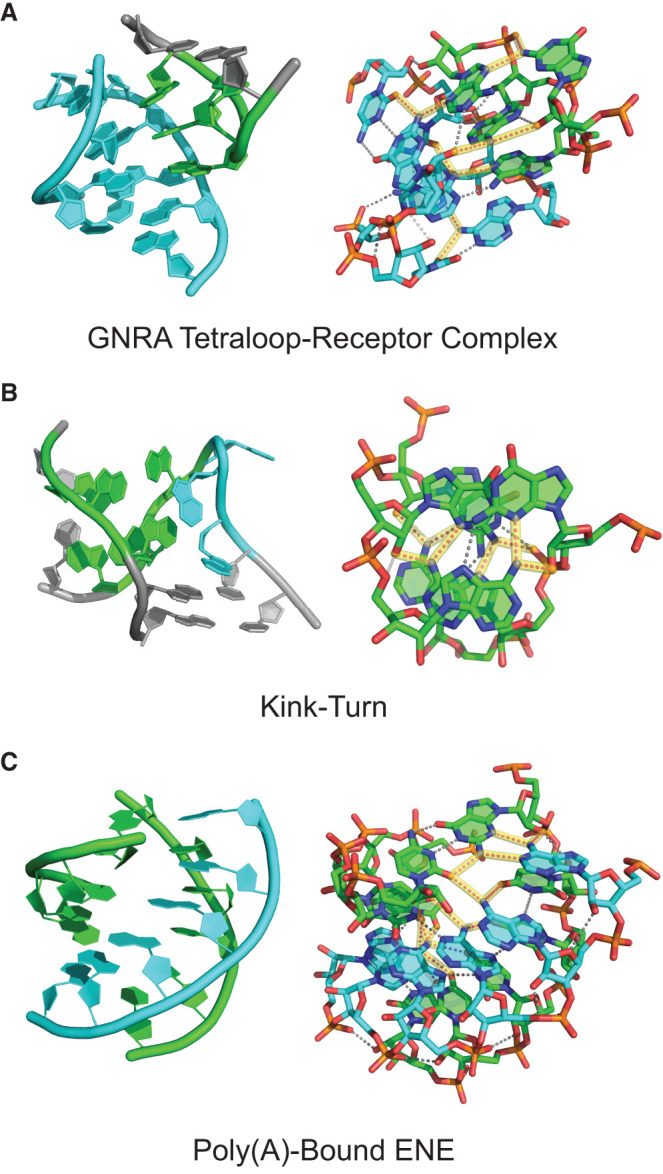
Structured RNAs enriched in dual-donating amines. (*A*) GNRA tetraloop receptor (cyan) bound to a GAAA tetraloop (green) from the P4–P6 domain with five dual-donating amines (PDB ID 1HR2). (*B*) Kink-turn from the SAM riboswitch with a 3 nt bulge (cyan) and series of sheared GA base pairs (green) with four dual-donating amines (PDB ID 2GIS). The stick representation on the *right* features only the sheared GA pairs. (*C*) Poly(A) strand (cyan) bound to the U-rich internal loop and GC closing base pair of an ENE (green) from a rice transposase mRNA with seven dual-donating amines (PDB ID 7JNH). For all panels, *left* is a cartoon representation, and *right* is a stick representation. Magenta dots highlighted in yellow depict H-bonds from dual-donating amines and gray dots depict other H-bonds. Cross-eye stereoviews are provided in Supplemental Figure S8, and the three structures are provided as Supplemental Structures S1–S3.

Dual-donating amines play essential roles in functional RNAs. Indeed, using manual curation, we found multiple dual-donating amines in a number of key RNA motifs. To appreciate their importance to RNA structure, we feature three such motifs here (Fig. 9, Supplemental Fig. S8 is stereo). The 11 nt GNRA tetraloop receptor contains an internal loop that binds a GNRA tetraloop via tertiary interactions (Juneau et al. 2001; Butcher and Pyle 2011). An example from the self-splicing group I intron reveals five dual-donating amines within the confines of this relatively small structure (Fig. 9A, Supplemental Fig. S8A is stereo). Two of the dual-donating amines correspond to the two most prevalent acceptor pair categories for G(N2) (Fig. 4B): One G(N2) is in the receptor and forms an A-minor motif with the GAAA tetraloop, donating to both the tetraloop and the receptor (Fig. 5E), while the other G(N2) is the first residue in the GAAA tetraloop, donating to A(N7)/N(NPO) (Fig. 5F); notably, a single A interacts with both of these G's. Additionally, one of the A dual-donating amines from the receptor H-bonds with an A(N1) from the tetraloop and a U(O2) from the receptor, corresponding to the third most prevalent acceptor pair for A(N6) (Fig. 4B). Remarkably, most residues in the receptor and the bound GAAA tetraloop are involved in dual-donating amine interactions, either as a donor, an acceptor, or both, emphasizing the prevalence of these interactions in tertiary structure.

Next, we feature the kink-turn motif in the *S*-adenosylmethionine (SAM) riboswitch, which involves a 3 nt bulge loop flanked on one side by three tandem sheared GA base pairs (Fig. 9B, Supplemental Fig. S8B is stereo; Montange and Batey 2006; Butcher and Pyle 2011; Lilley 2012). As the name suggests, the kink-turn motif results in a severe bend between the axes of the helices flanking the bulged loop. A crystal structure of the SAM riboswitch depicts four dual-donating amines in the three tandem sheared GA base pairs, with the central GA pair having two dual-donating amines and the terminal GA pairs each having one dual-donating amine. The A amines of these sheared GA pairs dual donate to G(N3)/N(O2′), which corresponds to the most prevalent acceptor pair category for A(N6) (Fig. 4B, 5A). These noncanonical base pairs hold the phosphate backbone of the opposing strands closer together, with interstrand phosphate distances of 12.9–16.0 versus 18–21 Å for a normal RNA helix (Supplemental Fig. S9; Pande and Nilsson 2008). With four dual-donating amines in the kink-turn, the density of these amines is like that in the tetraloop-receptor complex.

Finally, we present a structure of a poly(A)-bound element for nuclear expression (ENE) (Torabi et al. 2021). The poly(A) tail of an ENE-containing transcript binds the U-rich internal loop of the ENE to form an RNA triplex structure, which increases the half-life of an oncogenic Kaposi's sarcoma-associated herpesvirus (KSHV) transcript (Conrad and Steitz 2005; Mitton-Fry et al. 2010). Two ENEs—an upper and a lower—from a rice transposase mRNA are depicted in a crystal structure with a bound poly(A), revealing many interactions involving dual-donating amines (Fig. 9C, Supplemental Fig. S8C is stereo). Here, we focus on the U-rich internal loop and closing GC base pair of the upper ENE, complexed with the poly(A). Seven dual-donating amines span across this segment. Four contribute to contiguous UAU triplets of the U-rich internal loop, where the A(N6) dual donates to a U(O4)/U(O4) acceptor pair, the fourth most prevalent acceptor pair for A(N6) (Fig. 4B). The other three dual-donating amines participate in a quintuple-base interaction that is at the top of the U-rich internal loop and involves the closing GC base pair. This quintuple-base interaction contains a WC/H A-minor motif, which corresponds to the top acceptor pair for A(N6) (Figs. 4B, 5B). In sum, the GNRA tetraloop-receptor complex, kink-turn motif, and poly(A)-bound ENE, which are central to catalytic introns, riboswitches, and transcripts in cancer-causing viruses, display a remarkably high density of dual-donating amines. Apparently, dual-donating amines play a role in stabilizing and compacting diverse tertiary structure motifs in functional RNAs.

## DISCUSSION

We developed a cheminformatic pipeline for assessing the prevalence and context of dual-donating amines in RNA structure. High-resolution structures of RNAs, curated by the Representative Sets of RNA 3D Structures, were inspected for H-bonds from each of the two H's of exocyclic amines, using distance and angle criteria determined herein. We then classified nucleobase amines as non-, single-, or dual-donating and performed further analysis, including the consideration of noncanonical geometries. Importantly, this approach is reliant on experimental RNA structures deposited in the PDB; as such, our findings do not reflect any special characteristics of under- or nonrepresented RNAs.

Our study uncovered an enrichment of dual-donating amines in diverse base pairs including (1) *trans* AA pairs, which contribute to S-motifs, (2) A-minor interactions, which occur in GNRA tetraloop-receptor complexes, (3) sheared GA pairs, which are present in kink-turns, and (4) WC/H A-minor interactions, which occur in some ENE motifs (Figs. 5, 8, 9). Additionally, we identified dual-donating amines engaging with amino acids in the major and minor grooves (Supplemental Fig. S2).

We also found that the dual-donating amine motif is largely buried, with persistent high atom density and limited solvent accessibility (Fig. 6; Supplemental Fig. S3). Hosseini and colleagues reported reduced solvent accessibility of Gs in a mitochondrial ribosome small subunit (SSU) when compared to a bacterial ribosome SSU as a strategy to avoid oxidative damage (Hosseini et al. 2018). Our observations of the prominence for G(N2) to dual donate (Fig. 4A) and of the tendency for dual-donating amines to be buried are consistent with these findings. Intriguingly, the authors also showed that in both the bacterial and the mitochondrial ribosome SSUs, A's, C's, and G's had lower solvent accessibility when compared to U, which is less easily oxidized and lacks an exocyclic amine. Thus, on the one hand, the three nucleobases with exocyclic amines are more easily oxidized, but on the other hand, their amines provide a way to avoid oxidation through burial assisted by dual-donation.

One reason why dual-donating amines may play an outsized role in RNA structure is that RNA is replete in acceptors (Fig. 2). The fivefold excess of H-bond acceptors over donors makes the RNA backbone largely self-avoidant. Indeed, small molecules with multiple H-bond donors, such as urea with two amines, cause RNA to unfold (Shelton et al. 1999; Lambert and Draper 2012; Jaganade et al. 2019; Raghunathan et al. 2020). The abundance of H-bond acceptors makes a dual-donating amine capable of stitching together local RNA structures, as evidenced by turn motifs, including the kink-turn and S-motif, and long-range interactions, such as between the GNRA tetraloop and its receptor.

The strength of base-pairing may be enhanced by the dual-donating amine, which it can accomplish by reducing the conformational entropy loss for the RNA upon folding and by strengthening H-bonding. Forming H-bonds incurs an entropic penalty of loss of motion. Since the amine loses that entropy once in forming the first H-bond, it may not have to lose it again in forming the second H-bond, providing a reduction in conformational entropy loss of the RNA, favoring folding. We identified many diverse dual-donating amine motifs that could aid folding (Fig. 5). Some of these motifs may also have strengthened H-bonding, particularly those found in Figure 5C, D, and F and Supplemental Figure S2. In these cases, the dual-donating amine donates to a phosphate or a peptide backbone carbonyl. Notably, the phosphate NPO and carbonyl groups have estimated atomic charges of −0.78 and −0.57, respectively (Cornell et al. 1995). We highlight the interaction in Figure 5D as an example, where the C(N4) of a canonical GC base pair donates to a phosphate. This interaction should favor stronger electron donation by the amine of the C into the ring system, which would result in the development of greater positive charge on the N4 and negative charge on the N3 and O2. Charge development on these three atoms should move their p*K*_a_’s toward neutrality, which should strengthen each of the three H-bonds to the G due to better p*K*_a_ matching (Herschlag and Pinney 2018). Strong electron donation into the ring system may also perturb other chemical properties of the nucleobase, which could have implications for covalent modifications and chemical probing. Finally, knowledge of how dual-donating amines contribute to important structural motifs could aid RNA 3D structure prediction.

## MATERIALS AND METHODS

### Experimental structures and representative data set

#### Reducing redundancy in RNA-containing structures

While the PDB includes many RNA-containing entries, a number of these structures are highly similar to one another. For instance, a search on rcsb.org for structures containing a Polymer Entity Description matching “28S ribosomal RNA” or “28S rRNA” and the Scientific Name of the Source Organism matching “Homo sapiens” returned 179 results (as of May 28, 2025). The Representative Sets of RNA 3D Structures offers a well-documented and traceable solution to the reduction of RNA structural redundancy and includes a method of selecting representative RNA structures from groups of similar structures (Leontis and Zirbel 2012). The underlying algorithm updates the database weekly and considers all RNA-containing structures deposited into the PDB. It creates integrated functional elements (IFEs), which are units of one or more RNA chains. If two RNA chains from the same structure do not interact to an appreciable extent, they are assigned separate IFEs. For example, the 28S rRNA and 18S rRNA of PDB ID 8GLP, a structure of the 80S ribosome (Holm et al. 2023), belong to two separate IFEs because they interact relatively little with one another. Meanwhile, the 28S rRNA and 5.8S rRNA of this structure are assigned to the same IFE because there is sufficient interaction between the two chains. IFEs are sorted into equivalence classes, where they are grouped with other IFEs that are from the same species and exhibit similar structures. For instance, many IFEs containing the 16S rRNA of *Escherichia coli* ribosomes are grouped into the same equivalence class. A representative IFE for each equivalence class is selected by the Leontis and Zirbel (2012) method based on a calculated composite quality score that considers various metrics of the experimental structure; further details can be found at http://rna.bgsu.edu/rna3dhub/nrlist.

#### Data set used for this work

Our workflow considered the representative IFE from each equivalence class, using the 3.386 release (dated May 7, 2025) of the Representative Sets of RNA 3D Structures, referred to herein as the “Representative Data Set.” Several resolution thresholds were available, and we chose the 3.0 Å cutoff, which afforded 1832 high-quality RNA-containing structures and includes 2170 representative IFEs. Information on these IFEs was extracted from the full CSV file that was downloaded on August 22, 2025, using the API for the Representative Sets of RNA 3D Structures. Notably, our workflow analyzed 253,494 amines from this data set. While initially constructing our workflow, we worked with release 3.326 (dated March 13, 2024), which provided 197,765 amines from 1653 high-quality RNA-containing structures split into 1934 representative IFEs. Thus, there was a 28% expansion in the number of amines to analyze within just a little over a year's time.

Two aspects of our use of PDB entries are important to note. First, except for the hydrogens that are modeled using PyMOL, our workflow only evaluates the coordinates provided by each crystallographic information file for the PDB entries. Therefore, for each PDB entry, we consider only the asymmetric unit, which may not reflect the entire biological assembly. Second, when preparing a new release, the Representative Sets of RNA 3D Structures algorithm only considers a single version, often the first, of any given PDB entry. Meanwhile, unless they are provided manually, our workflow retrieves the most recent version of the PDB entries. Consequently, there are likely some changes between the structures considered by the database algorithm and those retrieved by our workflow. While noteworthy, we do not expect that these differences in PDB entry versioning to meaningfully impact our results. Version information corresponding to PDB entries that can be used to reproduce the data in this paper is reported in Supplemental Table S2.

#### Limitations

It is noteworthy that while the Representative Data Set has reduced redundancy, some still exists. For instance, there are 26 representative IFEs that contain the eukaryotic large subunit rRNA, with Rfam ID RF02543 (Ontiveros-Palacios et al. 2025), and that have 1000 or more observed nucleotides. This is because each of the 26 corresponding equivalence classes is associated with a different species. Furthermore, rRNAs make up much of the Representative Data Set on a per-residue basis. While only 7.8% of the IFEs contain one or more rRNA chains, 73.7% of the amines from all representative IFEs belong to such chains. Here, a chain was considered to be ribosomal based on the value in the “standardized_name” column within the full CSV file mentioned in the preceding section. Of course, even if all redundancy were removed, the data set would still only have those RNAs whose structures have been solved, reflecting the preferences of the structural biology community.

### Computational approach

#### The workflow

We created a computational workflow that uses Python to collect and process data obtained from the structures; additionally, the R programming language was used for data analysis and plotting. Key Python libraries used in the workflow include PyMOL (The PyMOL Molecular Graphics System, Version 3.0, Schrödinger, LLC.), Biopython (Hamelryck and Manderick 2003; Cock et al. 2009), and GEMMI (Wojdyr 2022). The Snakemake program was used to manage our workflow because it improves reproducibility and makes computational pipelines scalable, especially when used with high-performance computing (Mölder et al. 2021). The workflow can be found in its associated GitHub repository at https://github.com/The-Bevilacqua-Lab/dual-donating-amines.

#### Mining and identifying dual-donating amines

The workflow was used to iterate through each A, C, and G in the representative IFEs and identify H-bonds donated by their exocyclic amines. Each structure was retrieved from the PDB, any H's present were removed, and then H's were added to the amines of chains specified by the IFE using PyMOL. Rare problematic A, C, or G residues (e.g., a G(N2) not having two H atoms) were excluded from further consideration, totaling 17 residues in this case. Our code identified acceptors that reside within 4.1 Å of the amine nitrogen and that belong to polymers (e.g., RNA and proteins) but not to the parent nucleobase. The distance between each amine H and the nearby acceptor was then measured. Also, the angle between the amine nitrogen, each amine H, and the nearby acceptor was measured. Because each amine bears two H's, two distance and angle measurements were made for every identified amine-acceptor pair. No more than one of these two would form an H-bond with reasonable geometry (Fig. 1).

H-bond distance and angle criteria were determined after visualizing a heat map depicting distance and angle measurements of the amine-acceptor pairs from the Representative Data Set (Fig. 3A). Because each amine-acceptor pair contains two sets of measurements (one for each amine H), only the set with the greatest nitrogen–H–acceptor angle was included with this plot. The A(N6)–U(O4), C(N4)–G(O6), and G(N2)–C(O2) pairs were excluded owing to their prevalence in canonical base-pairing. Likewise, the putative G(N2)–C(N3) pair was excluded because of its proximity to the G(N2)–C(O2) H-bond found in GC base pairs. Importantly, these four amine-acceptor pairs were not excluded from other analyses. After establishing the H-bonding criteria, each amine-acceptor pair was designated as H-bonding if the H–acceptor distance and the nitrogen–H–acceptor angle met the criteria.

Nucleobase amines were classified as non-, single-, or dual-donating (Fig. 1). For each dual-donating amine, the pair of acceptor atoms that participate in the H-bonding interactions was identified. In cases where a dual-donating amine H donates to two or more acceptors, only the acceptor that resulted in the greatest H-bond angle was included as a member of the amine's acceptor pair.

#### Canonical GC base pairs

We occasionally considered the data in the context of GC base-pairing when a G or C was involved in the dual-donating amine interaction. Canonical GC base pairs were considered to be present when both the G(N2)-to-C(O2) and C(N4)-to-G(O6) H-bonds were formed.

#### Visualizing instances of dual-donating amines using PyMOL

Our workflow created 15 CSV files that list all identified cases related to the interactions depicted in Figure 5 and Supplemental Figure S2 and three CSV files that list cases corresponding to Location 1 and Location 2 of Figure 8C. A Python script named “review_amines.py” is included with the workflow available from the GitHub repository. The 18 CSV files were used as inputs to review_amines.py, creating 18 corresponding Python files that are intended to be run using PyMOL. We provide all 36 of these files in the Supplemental Material along with brief descriptions in Supplemental Table S1. Use of PyMOL with these Python files enables visualization of all the instances of dual-donating amines listed in the original CSV files. Instructions on how to use these 18 Python files are provided in the README.md file in the Supplemental Material.

#### Solvent accessible surface area calculations

Biopython was used to calculate solvent accessible surface area (SASA) on the nitrogens of the A, C, and G amines (Hamelryck and Manderick 2003; Cock et al. 2009). For each RNA-containing structure downloaded from the PDB, the code loads the structure and inspects it for the presence of H's. If any are present, the structure is saved to a new file without them, and then the new structure is loaded. The SASA of each nitrogen is calculated based on the method of Shrake and Rupley (1973) with the default parameters provided by Biopython, including a probe radius of 1.40 Å.

#### Heavy atom density calculations

The densities of heavy (i.e., non-H) atoms within two regions of interest (ROIs), which encompass each A, C, and G amine nitrogen, were calculated (Fig. 6B). First, the code worked with PyMOL to count the number of heavy atoms from polymers (e.g., RNA and proteins), as defined by PyMOL, within 7.5 and 12.5 Å of each nitrogen. The density in ROI 1 was then calculated by dividing the number of heavy atoms ≤7.5 Å from the amine nitrogen by the volume of a sphere with a radius of 7.5 Å. For ROI 2, the two heavy atom counts were subtracted to find the number of heavy atoms >7.5 and ≤12.5 Å from the amine nitrogen. The density was then determined by dividing this number by the difference in volumes of two spheres with radii of 7.5 and 12.5 Å.

#### Dihedral measurements

The values of the χ, η, and θ RNA dihedrals were calculated to study nucleobase orientations relative to their attached sugars (χ dihedral) and sugar-phosphate backbone conformations (η and θ dihedrals). For the χ measurements, PyMOL was used to acquire the dihedral along the O4′, C1′, N9, and C4 atoms for purines and along the O4′, C1′, N1, and C2 atoms for pyrimidines (Bloomfield et al. 2000). The η and θ dihedrals are pseudotorsions that involve the P and C4′ atoms of the residue in question and its immediate neighbors. These measurements were obtained using the NaTorsion program from the AMIGOS III repository (Shine et al. 2022).

#### Calculation of P-values

The SASA distributions of non-, single-, and dual-donating amines were compared using pairwise Wilcoxon rank sum tests, and the resulting *P*-values were adjusted to control the false discovery rate (FDR) (Supplemental Fig. S3A; Benjamini and Hochberg 1995). This approach was also used to compare the distributions of heavy atom densities (Fig. 6C; Supplemental Fig. S3C). For the distributions of χ dihedrals, which are circular, Watson's test for homogeneity was used. Because this test generated *P*-value ranges (not discrete *P*-values), control for FDR was not performed (Supplemental Fig. S4). All calculations were made using the R programming language.

#### Clustering analysis

The η and θ pseudotorsions were plotted as heat maps for non-, single-, and dual-donating amines to reveal differences between them (Fig. 8A–C), and locations with higher counts in the heat maps for dual-donating amines were identified. Some of the residues in these locations were visualized manually using PyMOL and appeared to be involved in S-motifs. Residues from each of the identified locations that correspond to a top acceptor pair category/categories (Fig. 8D) were selected for further study, as specified in the Results. For each of these residues, a 6 × 5 nucleotide fragment was extracted from the parent structure containing the residue of interest and other nearby residues that potentially make up an S-motif. Fragment alignments and clustering analyses were then carried out to better understand the extent to which residues in the identified locations are involved in S-motifs.

For fragment alignments of each of the locations identified above, we performed RMSD calculations between all the fragments using Biopython's Superimposer module (Hamelryck and Manderick 2003; Cock et al. 2009), and an RMSD distance matrix was constructed. To ensure equal numbers of atoms were present within fragments of different sequences, a coarse-grained approach was adopted in which each nucleotide was represented using five atoms: P, C4′, N9, C2, and C6 for purines, and P, C4′, N1, C2, and C4 for pyrimidines (Ramakers et al. 2024). Hierarchical clustering analyses were then performed using the scikit-learn library (Pedregosa et al. 2011). To assess potential RMSD cutoffs for sorting fragments into clusters, we calculated three clustering validation metrics: the Silhouette score, the Calinski–Harabasz index, and the Davies–Bouldin index (Supplemental Fig. S5). These metrics were calculated across a range of RMSD cutoffs determined from the linkage matrix. The smallest cutoff was the value where at least one cluster with four or more members was formed, and the largest cutoff was the integer below the maximum distance present.

The optimal RMSD cutoff was chosen from the Silhouette score because it resulted in a more manageable number of clusters. The cluster with the most members was designated as the dominant cluster (Supplemental Fig. S6). Within each of the resulting clusters, a representative structure was selected, which was done by finding the cluster's average structure, calculated as the mean of all coarse-grained atomic coordinates within the cluster, and then choosing the structure with the smallest RMSD to it (Fig. 8E; Supplemental Fig. S7).

## SUPPLEMENTAL MATERIAL

Supplemental material is available for this article.

## Figures and Tables

**Andrew J. Veenis:**
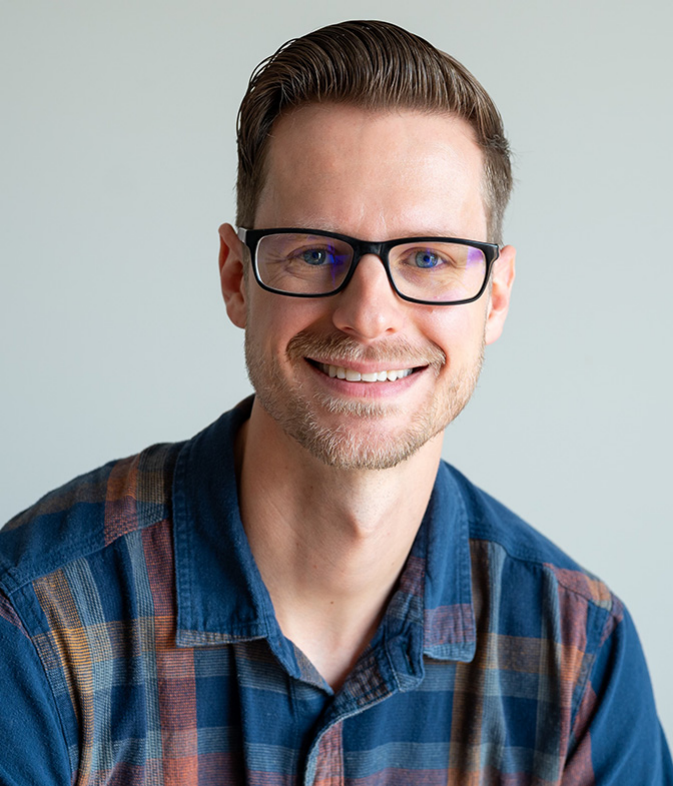


## MEET THE FIRST AUTHOR

***Meet the First Author(s)* is an editorial feature within *RNA*, in which the first author(s) of research-based papers in each issue have the opportunity to introduce themselves and their work to readers of *RNA* and the RNA research community. Andrew J. Veenis is the first author of this paper, “Prevalence of dual-donating amines in key regions of functional RNAs.” Andrew is a Postdoctoral Scholar at Penn State in the laboratory of Dr. Philip Bevilacqua. His research primarily involves using computational chemistry and informatics approaches to study noncovalent interactions in structured RNAs and p*K*_a_ shifting of RNA residues.**

**What are the major results described in your paper, and how do they impact this branch of the field?**

Our study provides a survey of how dual-donating amines are involved in structured RNAs. In a biopolymer dominated by H-bond acceptors, these interactions provide a means to help stitch together different regions of the RNA, promoting structure and ultimately function. Distinct RNA folds are stabilized by a balance of diverse noncovalent interactions, and these results enrich our understanding of how H-bonds contribute to RNA structure.

**What led you to study RNA or this aspect of RNA science?**

I rotated through multiple research labs at the start of graduate school, ending with the Bevilacqua lab. While I had never studied RNA, the research in this lab sounded interesting, and Dr. Philip Bevilacqua was recommended as being an excellent mentor. During my rotation, I was impressed by how engaged Phil was with his lab members and decided to join his group. My passion for RNA research then grew as I learned about the many biological functions of RNA and the underlying chemistry that drives those functions.

**During the course of these experiments, were there any surprising results or particular difficulties that altered your thinking and subsequent focus?**

The core of this study centers on the residues directly involved in dual donor interactions. While this focus was key to establishing a foundation for understanding these interactions, I wanted to use our computational workflow to identify broader structural motifs comprising dual-donating amines. The work from Dr. Anna Pyle's lab inspired me to compare the η and θ pseudotorsions between non-, single-, and dual-donating amines, and I was excited to find the two locations in the resulting plots that revealed enrichment in residues bearing dual-donating amines. This led to the excellent clustering analyses by coauthor Dr. Sharear Saon and verification of the S-motif as being the key structural motif relevant to the two locations.

**What are some of the landmark moments that provoked your interest in science or your development as a scientist?**

When I was a graduate student, I struggled with my dissertation research and experienced substantial burnout. My dad had recently started a plastic bottle manufacturing business, and this seemed to be an excellent opportunity to do something different. With the support of Phil and my family, I completed my dissertation, graduated, and was hired as an engineer to work for my dad's company. While my roughly two-year stint in manufacturing introduced me to some fascinating technologies and presented exciting problems to solve, one issue became clear: I missed research. Phil and I eventually had a conversation about continuing some work on dual-donating amines that we began during my graduate studies. This led to my postdoctoral role in his lab and the publication of this paper. Doing research again confirmed to me that being a scientist is a central part of my identity. I look forward to continuing my career journey, where I will contribute to our expanding knowledge of how our world works.

**If you were able to give one piece of advice to your younger self, what would that be?**

I would advise my younger self to write early. Much of my research in graduate school entailed running simulations of the hairpin ribozyme to estimate the p*K*_a_ of its nucleophilic alcohol. At one point, I started to spend some time extending my methodology to the *glmS* ribozyme. When I put together an outline, I realized that this broadening to the *glmS* ribozyme was a digression, and that I needed to maintain focus on studying the hairpin ribozyme. Writing a draft earlier in my research process would likely have saved me valuable time.

**Are there specific individuals or groups who have influenced your philosophy or approach to science?**

I attended Eastern Mennonite High School in Virginia, where I completed multiple biology classes taught by Mr. Myron Blosser. This is when my passion for the biological sciences sparked. While providing us a foundation of what was known, Mr. Blosser had a knack for captivating students by helping us realize all the discoveries that had yet to be made. Taking his classes made me excited to learn more about biology, and this excitement is something that all scientists should seek throughout their career. Find a subject that naturally makes you want to learn more and pursue that line of research.

**What are your subsequent near- or long-term career plans?**

I intend to transition into a role where I can practice applied science to develop new technologies, which will likely entail working in the biotech or pharma sector. While keeping my options open, I aim to secure a job that involves both computation and chemistry.
